# Evaluating the hepatitis B vaccination impact in the Republic of Moldova: A nationwide representative serosurvey of children born in 2013

**DOI:** 10.1016/j.ijregi.2023.11.003

**Published:** 2023-11-08

**Authors:** Michael Brandl, Alexei Ceban, Octavian Sajin, Victoria Bucov, Alina Cataraga, Silvia Stratulat, Nicolae Furtuna, Veaceslav Gutu, Stela Gheorghita, Martyna Gassowski, Liudmila Mosina, Antons Mozalevskis, Sandra Dudareva, Siddhartha Sankar Datta

**Affiliations:** 1 Robert Koch Institute, Dept. of Infectious Disease Epidemiology, Berlin, Germany; 2 Charité – Universitätsmedizin Berlin, corporate member of Freie Universität Berlin and Humboldt-Universität zu Berlin, Berlin, Germany; 3 WHO Country Office in the Republic of Moldova, Chisinau, Republic of Moldova; 4 National Agency for Public Health, Chisinau, Republic of Moldova; 5 WHO Regional Office for Europe, Copenhagen, Denmark; 6 WHO Headquarters, Geneva, Switzerland

**Keywords:** Hepatitis B, Vaccination, Europe, Republic of Moldova, Seroepidemiologic study, Prevalence

## Abstract

**Objectives:**

The WHO European Region set targets for the control of hepatitis B through immunization, including prevalence of hepatitis B surface antigen (HBsAg) at ≤0.5% in vaccinated cohorts. The Republic of Moldova implemented universal hepatitis B vaccination since 1995. We conducted a nationwide representative serosurvey to estimate HBsAg seroprevalence in children born in 2013 to validate hepatitis B control targets.

**Methods:**

We used probability-based sampling and a two-stage cluster design. All children born in 2013 and registered in primary healthcare facilities were eligible for participation. We tested blood samples of all participants for hepatitis B core antibody (anti-HBc), using Enzyme-Linked Immunosorbent Assay (ELISA). Anti-HBc-positive samples were tested for HBsAg and HBsAg-positive samples confirmed, using ELISA. We obtained information on hepatitis B vaccination from vaccination cards.

**Results:**

Of 3352 sampled children, 3064 (91%) participated. Most participating children were 7 years old (n = 3030, 99%), 1426 (48%) were girls. The weighted, national seroprevalence estimate was 3.1% (95% confidence interval = 2.1-4.5) for anti-HBc and 0.21% (95% confidence interval = 0.08-0.53) for HBsAg.

**Conclusion:**

The study demonstrated the impact of hepatitis B vaccination and allowed the Republic of Moldova to validate regional hepatitis B control targets. Other countries with high vaccination coverage could use hepatitis B serosurveys and apply for validation. Sustained efforts in the Republic of Moldova will be crucial on the path to hepatitis B elimination.

## Highlights

• First study in the Republic of Moldova assessing regional progress towards hepatitis B control targets
• The study documented the impact of the hepatitis B vaccination programme in the Republic of Moldova
• The Republic of Moldova drastically reduced the prevalence of hepatitis B in vaccinated cohorts

## Introduction

Vertical transmission from mother to child (MTCT) and horizontal transmission at very young age are the key drivers of the burden of hepatitis B worldwide [1]. The rate at which hepatitis B virus (HBV) infections become chronic is around 90% in new-borns but decreases with age to about 20–30% in children, with still no cure available and lifelong medication being the only treatment option [2,3]. Hepatitis B vaccines have been shown to be safe and effective, and administration of hepatitis B birth dose, followed by either two or three additional doses as part of the childhood vaccination schedule during the first year of life, is likely to provide lifelong protection [4,5].

In 2016, the World Health Organization (WHO) published the first Global Health Sector Strategy on Viral Hepatitis 2016-2021 (GHSS) to tackle global HBV infections, morbidity, and mortality [6]. The GHSS set ambitious targets for the years 2020 and 2030, including reduction of incidence and mortality, high hepatitis B vaccination coverage, and prevention of mother-to-child transmission (PMTCT). It stated the overarching aim to eliminate hepatitis B as public health threat by 2030 [7]. High and sustained vaccination coverage is essential to prevent HBV transmission and critical to the foundation for hepatitis B control and elimination [8].

The WHO European Region, where an estimated 14 million people live with chronic hepatitis B, translated the GHSS into a reginal action plan, adopted by the Regional Committee in 2016, in order to accelerate the response to the viral hepatitis epidemics and contribute to regional and global viral hepatitis elimination efforts [9,10]. The action plan identified PMTCT as a key aspect of hepatitis control and set three regional control targets for the European Region: 90% coverage with measures to prevent perinatal transmission, including timely birth dose (within 24 hours of birth), 95% coverage with three-dose hepatitis B vaccine for children, and maximum 0.5% of hepatitis B surface antigen (HBsAg) prevalence in vaccinated cohorts [9]. The target of HBsAg seroprevalence is assessed through serosurveys, which are the principal method to ascertain the impact of vaccination, whereby a representative population is sampled to determine the prevalence of serological markers [11].

The Republic of Moldova, located in the Eastern part of the WHO European Region, has an estimated population of 2.6 million inhabitants, of which about 42% live in urban areas [12]. The country committed to the control of viral hepatitis by developing and implementing its first national viral hepatitis programme in 1997 and is already at its fourth programme for the years 2017-2021 [13,14]. Since 1966, incidence of HBV infection has been reported by the Republic of Moldova and it was considered a highly endemic country, but reliable disease prevalence estimates were scarce [15]. In 1994, a serological study among 158 first graders (mean age 7 years) found a proportion positive for HBsAg of 10.8% [16].

After the introduction of selective immunization of new-borns against HBV in the Republic of Moldova in 1989, the incidence of acute hepatitis B has been declining, especially after the implementation of hepatitis B vaccination in the routine immunization programme in 1995 [15]. Coverage with three doses of hepatitis B vaccine (HepB3) has been above 80% since 1996, and the coverage with hepatitis B birth dose above 90% since 2000, according to the WHO/UNICEF Joint Reporting Form on Immunization [17]. With high prevalence of HBsAg in adult populations in the Republic of Moldova, timely birth dose and other PMTCT measures are crucial to protect new-borns from infection with HBV [15,16]. To date, no representative study among children born after the introduction of the national hepatitis B vaccination programme in the Republic of Moldova has been conducted.

In a collaborative effort, the National Agency for Public Health, the Ministry of Health of the Republic of Moldova, the WHO Regional Office for Europe, and the Robert Koch Institute designed and implemented a serosurvey in 2020 to determine the HBsAg prevalence among children born in 2013 and document the impact of hepatitis B vaccination in the Republic of Moldova.

## Methods

### Study design

We conducted a cross-sectional population-based representative serosurvey in the Republic of Moldova among children born in 2013. For this study, we used a stratified two-stage cluster design.

### Study population

Children born in the Republic of Moldova in 2013, registered in primary health care facilities (HCF), and attending the first school grade in 2020/2021 were eligible for participation. Parents or legal guardians of participating children were present during the sample collection and provided written informed consent. There were no a-priori exclusion criteria.

### Sample size

Based on national hepatitis B surveillance data and vaccination coverage data for hepatitis B vaccine in the Republic of Moldova since 2000, the prevalence in vaccinated cohorts was expected to be low and was set at 0.30% for the purpose of sample size calculation. To ensure a feasible overall sample size, we set the upper precision bound for the national level at 0.69%. The sample size (n) for a one-sided test with the significance level α and power 1-β was computed using the following formula:

$$n={\left(\frac{z_{1-\alpha }\sqrt{p_{0}\left(1-p_{0}\right)}\;+\;z_{1-\beta }\sqrt{p_{1}\left(1-p_{1}\right)}}{\delta }\right)}^{2}$$

$p_{0}$ = expected prevalence, $p_{1}$ = alternative prevalence (upper bound), $z_{1-\alpha }$ = quantile of the standard normal distribution, δ = difference between expected and alternative prevalence ($p_{1}$ - $p_{0}$), also called effect size. The α-level was set at 0.05 and the power (1-β) at 80%.

The design effect was set at 2 and was accounted for in a second step of the calculations. This resulted in a total sample size of 3352 children.

### Study location and stratification

The study took place in both rural and urban settings throughout all districts and municipalities of the right part of the Dniester River in the Republic of Moldova. Blood sample collection and data extraction from patient files took place at HCF.

We stratified the study sample by geographical region into two study areas:

Area 1: Chisinau municipality, which was not further divided, also referred to as a region.
Area 2: Remaining districts in the right part of Dniester River of the Republic of Moldova, divided into three regions: Central, North, and South.

We distributed the overall number of children across the two study areas and sampled 1246 children from Chisinau municipality and 2106 children from study area 2, corresponding to upper precision bounds of 0.99% and 0.81%, respectively (Appendix, Table A1). The number of children was larger in study area 2 in order to consider higher variability between the districts in the three regions.

We sampled numbers of children from each of the four regions as per population distribution of children born in the Republic of Moldova in 2013. Additionally, in order to ensure that the selected sample proportionally reflected the distribution of the target population by the residential location, HCF were stratified by their location (rural or urban). This resulted in a total of eight strata in the four regions.

Each HCF was designated as rural or urban based on the official classifications in the Republic of Moldova. It was observed that, e.g., some referral areas of urban HCF included villages located around the city limits and they did not conform to the official classification of the location. In order to avoid any misrepresentation of the residential location of the study participants, the study nurse or doctor recorded the residential location (urban or rural) of the child during the enrolment in addition to the information assigned to each HCF by location used in the survey.

### Sampling

We used probability-based sampling in two stages and set the cluster size at 30 children per cluster. The number of clusters sampled from each stratum was proportional to the size of the target population in the respective stratum. This resulted in a total of 116 clusters across the eight strata.

In the first sampling stage, we sampled HCF in each stratum which provide health care to children across the right part of Dniester river of the Republic of Moldova. We sampled HCF randomly with replacement and used probability proportional to the size of the registered population. This means, each HCF had a probability to be selected proportional to the number of children registered at the respective HCF and each HCF could be selected multiple times.

In each of the HCF, one doctor or department per cluster was selected, depending on the size and type of the facility. Then, 30 children born in 2013 who were registered with the selected doctor or department were identified using systematic random sampling, e.g. every second child from provided patient lists. If less than 30 children were identified, another doctor or department was randomly selected and this process was repeated until the target sample size was reached. Doctors then invited selected children for blood tests, provided their parents or legal guardians with the study information letter, and presented the informed consent form for signature at the blood test. If children, parents, or legal guardians refused participation, the next or a child who was skipped from the provided lists were included in the sample and invited.

### Laboratory testing procedure and algorithm

All samples were tested at the National Agency for Public Health of the Republic of Moldova for total antibody to hepatitis B core antigen (anti-HBc), using Bio-Rad Enzyme-Linked Immunosorbent Assay (ELISA) test kits (sensitivity 99.5%, specificity 99,5%). Samples positive for anti-HBc were tested for HBsAg and hepatitis B surface antibody (anti-HBs), using Dia.Pro ELISA test kits (sensitivity 98.8%, specificity 100%). HBsAg-positive samples were then tested with confirmatory Murex® ELISA test kits (sensitivity 100%, specificity 100%). We considered samples positive in confirmatory ELISA as acute or chronic infections with HBV. A detailed testing algorithm can be found in Appendix, Figure A1 and interpretation of serological test results in Appendix, Table A2.

### Data collection

The survey was conducted between December 2020 and January 2021. We collected demographic data on age, sex, region, and residential location of all participating children in the survey. Information on hepatitis B vaccination history was collected from vaccination records. We extracted data on vaccination status and dates of administration for hepatitis B birth dose, first dose, second dose, and third dose from the available vaccination records. Laboratory data were collected according to the testing algorithm and included results for anti-HBc, anti-HBs, and HBsAg. All children were asked, if they had family contact with a case or carrier of HBV.

### Data analysis

We described the study population and the confirmed HBsAg-positive cases by age, sex, region, residential location, and documentation of birth dose and three doses of hepatitis B vaccine. For the calculation of the timely administration of the birth dose, we excluded children with missing information on either date of birth or date of birth dose vaccination and where the date of vaccination was implausible.

We analysed laboratory testing results according to the four possible outcomes of the testing algorithm. Results are presented as absolute values and proportions with 95% confidence intervals (CI). The sampling process was self-weighted in regards of selection of children within strata. We used post-stratification weighting to correct for levels of non-response and the population distribution in the various strata. Additionally, we specified cluster identifiers in the analysis to adjust for the impact of the study design on the precision of the HBsAg prevalence estimates. Subjects belonging to a particular cluster tend to have characteristics that make them more similar to each other than to subjects from other clusters, which adds variability to the sample and was thus accounted for. We calculated the design effect, which is a measure for this increased variance due to clustering.

## Results

### Study population

**Figure 1:**
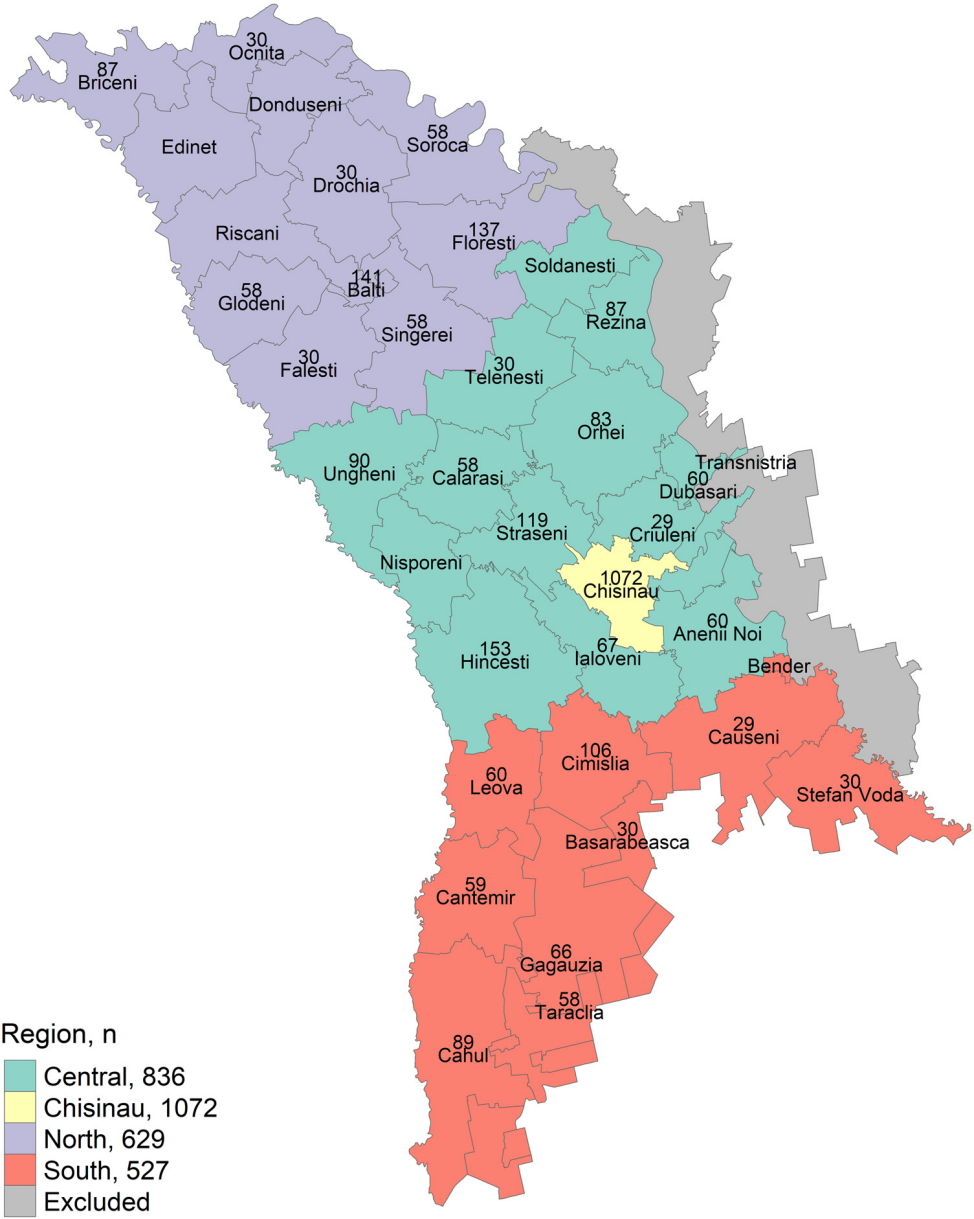
Numbers of reached study participants (n) per district and region in the Republic of Moldova, 2020/2021.

Out of a target population of 42,373 children, 3352 children were sampled and 3064 (91%) participated in the study. Signed informed consent forms were obtained from the parents or legal guardians of each of the participants. In study area 1, Chisinau municipality, 86% (1072/1246) of the targeted sample size could be reached and in study area 2 94% (1992/2108). Details on proportions of target sample size reached across the eight strata are presented in Table 1 and the geographical distribution of participating children across districts and regions is presented in Figure 1. Proportions above 100% relate to assignment of HCF as per the residential location, as explained in the methods.

**Table 1:** Number of targeted and reached participants and proportion of target sample size reached by strata (region and residential location), Republic of Moldova, 2020/2021.

Strata	Target number of participants	Number of participating children within sample	Proportion of target sample size reached
Chisinau urban	1117	925	83%
Chisinau rural	129	147	114%
North urban	412	379	92%
North rural	323	250	77%
Central urban	374	461	123%
Central rural	452	375	83%
South urban	291	265	91%
South rural	256	262	102%
**Total**	**3352**	**3064**	**91%**

### Descriptive results

Children who participated in the study were predominantly 7 years old (n = 3030; 99%) and male (n = 1604; 52%). Most participants were from Chisinau (n = 1072; 35%). Detailed characteristics of the study population and the HBsAg-positive cases are provided in Table 2.

**Table 2:** Characteristics of all study participants and confirmed HBsAg-positive participants, Republic of Moldova, 2020/2021.

Variable	Value	All participants (n = 3064)		HBsAg positive participants (n = 7)	
		n	%	n	%
Age (in years)	6	28	0.9%	0	
	7	3030	99%	7	100%
	8	6	0.2%	0	
Sex	Female	1460	48%	2	29%
	Male	1604	52%	5	71%
Region	Chisinau	1072	35%	2	29%
	Central	836	27%	3	43%
	North	629	21%	2	29%
	South	527	17%	0	
Residential location	Rural	1034	34%	0	
	Urban	2030	66%	7	100%
Hepatitis B birth dose	Available documentation of vaccination	2848	93%	5	71%
	No documentation of vaccination	216	7.0%	2	29%
Time of administration of birth dose	On the day of birth	1966	73%	3	60%
	1 day after birth	499	19%	2	40%
	More than 1 day after birth	227	8.4%	0	
	Missing or doubtful date	(372)		(2)	
Hepatitis B first dose	Available documentation of vaccination	2835	93%	5	71%
	No documentation of vaccination	229	7.5%	2	29%
Hepatitis B second dose	Available documentation of vaccination	2818	92%	5	71%
	No documentation of vaccination	246	8.0%	2	29%
Hepatitis B third dose	Available documentation of vaccination	2632	86%	5	71%
	No documentation of vaccination	432	14%	2	29%

HBsAg: hepatitis B surface antigen.

Complete information on the dates of hepatitis B vaccinations was available for 93% for birth and first dose, 92% for second dose and 86% for third dose. Among children for which full and plausible information on administration of the birth dose was available, 73% have received it on the day of birth and 19% on the next day. Thus, 92% of administered birth dose of hepatitis B vaccine in the surveyed population was within 1 day after birth.

Among the study participants, 99 (3.2%) tested positive for anti-HBc. In further testing of these samples, 52 (1.7%) were positive for anti-HBs and 15 (0.49%) for HBsAg. Seven (0.23%) HBsAg-positive samples were confirmed as positive with Murex® confirmatory ELISA.

All seven confirmed HBsAg-positive cases were 7 years old and five of them were males. Three of these cases were from Central region and two each from Chisinau and North regions – all cases were geographically related to urban HCF. In regions Central and North, two cases each were found in the same HCF, while the remaining three cases were found in different HCF.

Five cases had received a birth dose and three additional doses of hepatitis B vaccine. All five had received their birth dose vaccination within 1 day after birth (three on the same day, two on the next day). For the remaining two cases, no documentation on vaccinations was available. None of the seven cases stated that they had family contact with a known case or carrier of HBV.

### Weighted analysis of hepatitis B seromarkers

Hepatitis B seromarker results according to the four possible outcomes of the testing algorithm are displayed in Table 3. We found a weighted, national seroprevalence estimate of 3.1% (95% CI = 2.1-4.5) for total anti-HBc. Further testing of these samples resulted in estimated overall seroprevalence of 0.21% (95% CI = 0.08-0.53) for HBsAg, 1.6% (95% CI = 1.0-2.6) for anti-HBc plus anti-HBs, and 1.3% for anti-HBc only (95% CI = 0.9-1.8). Weighted proportions did not differ statistically from crude proportions after adjusting for population size and response rate with post-stratification weights.

**Table 3:** Analysis of laboratory test results in crude analysis and stratified analysis incorporating cluster effects, Republic of Moldova, 2020/2021 (n = 3064).

Laboratory test results	n	Crude proportion	95% Confidence interval	Weighted proportion	95% Confidence interval
Anti-HBc (+), anti-HBs (-), HBsAg (+)	7	0.23%	0.11-0.48%	0.21%	0.08-0.53%
Anti-HBc (+), anti-HBs (+), HBsAg (−)	52	1.7%	1.3-2.2%	1.6%	0.97-2.6%
Anti-HBc (+), anti-HBs (−), HBsAg (−)	40	1.3%	0.96-1.8%	1.3%	0.87-1.8%
Anti-HBc (−)	2965	96.8%	96.1-97.3%	96.9%	95.5-97.9%

Anti-HBc: hepatitis B core antibody, anti-HBs: hepatitis B surface antibody, HBsAg: hepatitis B surface antigen.

Weighted proportions of confirmed HBsAg prevalence stratified by sex, region, residential location, and study area are displayed in Table 4. Results did not differ statistically between the two study areas for which statistical power was pre-defined and sufficient. The design effect in the final analysis was 1.49 overall, 1.06 in Chisinau municipality, and 1.54 in the remaining territories. The higher design effect outside of Chisinau is explained by the distribution of cases across HCF.

**Table 4:** Stratified results of weighted analysis of HBsAg-positive participants, Republic of Moldova, 2020/2021 (n = 3064).

Variable	Value	Weighted HBsAg-positive proportion	95% Confidence interval
Sex	Female	0.12%	0.03-0.45%
	Male	0.30%	0.10-0.88%
Region	Chisinau	0.19%	0.05-0.81%
	Central	0.29%	0.08-1.08%
	North	0.29%	0.04-2.29%
	South	0	
Residential location	Rural	0	
	Urban	0.34%	0.16-0.72%
Study area	Chisinau municipality	0.19%	0.05-0.81%
	Remaining districts in the right part of Dniester River of the Republic of Moldova	0.22%	0.07-0.69%
Total		**0.21%**	**0.08 – 0.53%**

HBsAg: hepatitis B surface antigen.

## Discussion

The Republic of Moldova is one of the first countries in the WHO European Region that conducted a representative population-based HBsAg serosurvey in a vaccinated cohort in order to measure the impact of the hepatitis B vaccination programme [9]. The proof of a very low seroprevalence among children contributed to validation of reaching the hepatitis B control targets in the Republic of Moldova [9,18]. In our study, seven children (0.21%, 95% CI = 0.08-0.53) were found to be HBsAg positive, i.e., infected with HBV most likely acquired during or after birth. This result demonstrates the substantial impact of hepatitis B vaccination on chronic infections with HBV compared to data in the Republic of Moldova in 1994, when the seroprevalence among children in the same age was 10.8% [16]. In 1994, the children who were tested had not benefitted from the hepatitis B birth dose and subsequent childhood vaccinations.

There are few other countries in the WHO European Region that have demonstrated similar results after introduction of hepatitis B vaccination among children. A non-representative serosurvey from 2017 in the neighbouring country of Ukraine on 4596 children born between 2006 and 2015 also found a proportion positive for HBsAg of 0.2% [19]. In Georgia which is one of seven countries where pilots for the validation of elimination of hepatitis B and C were conducted, the HBsAg prevalence was estimated to be less than 0.1% among under 5-year-olds based on results from a serosurvey conducted in 2021 among 5-17-year olds [20]. In 1-6-year-old children in Tajikistan, born when the coverage with three doses hepatitis B vaccine was already over 80%, the seroprevalence in 2010 was estimated to be 0.4% (0.1-1.3%) [21]. All three of these countries from the region of Eastern Europe and Central Asia (EECA) in the WHO European Region introduced national vaccination programmes around the same time as the Republic of Moldova [22]. Additional countries in the WHO European Region outside EECA, where HBV prevalence is historically low, conducted serosurveys among adult or general populations and Italy and the Netherlands were the first countries within the region to receive validation for achieving regional hepatitis B control targets in 2020 [23].

In our study, five of the seven positive cases were boys. The risk of MTCT of HBV during birth may not differ between genders, but males have been associated with higher susceptibility to acute infection and an increased risk for persistent HBV infections [24]. Although our study lacks the power to measure the difference in prevalence between boys and girls, data from earlier studies suggest that boys develop lower levels of antibody titres after vaccination compared to girls [25,26]. This might lead to the situation that boys are more likely to get infected despite timely and completed hepatitis B vaccination. In our study, among HBsAg-positive children, three of the five boys and both girls were fully vaccinated against HBV, while there were no documented vaccinations for two of the boys. It was out of the scope of this study to confirm if the infections of the HBsAg-positive cases were acquired at birth or through contact with other HBsAg-positive cases like household contacts later in childhood.

All seven cases were found linked to urban HCF while only one case lived in a rural setting. In the sensitivity analysis, we analysed data according to this recorded residential setting in the dataset and did not observe any discernible differences in prevalence estimates (Appendix, Table A3). However, vaccination coverage for children in urban areas was lower for birth dose and for the third dose compared to children residing in rural areas (Appendix, Table A4). This is in line with sub-national immunization coverage data which the Republic of Moldova reports annually through the WHO/UNICEF Joint Reporting Form on Immunization. This is also in line with data reported by other Central Asian, Caucasian, and Eastern European countries and demographic and health surveys conducted in Kyrgyzstan and Tajikistan [27,28]. The Ministry of Health should address immunization equity with a special focus on increasing vaccination uptake in urban settings in the Republic of Moldova within the domain of the European Immunization Agenda 2030 [29].

Overall, we found a weighted proportion of 3.1% anti-HBc among 7-year-old children in the Republic of Moldova indicating exposure to HBV. In total, 1.6% were also positive for anti-HBs indicating resolved infection and 1.3% were isolated anti-HBc positive [30]. The beforementioned study in Ukraine, which showed similar levels of HBsAg, found 1.8% of anti-HBc-positivity without testing for anti-HBs [19]. A study in China among vaccinated hospitalized children found a proportion of 5.7% anti-HBc-positive children among 1-10 year-olds [31]. Eleven percent of anti-HBc-positive and HBsAg-negative children in our study had incomplete documentation of birth dose vaccination (compared to 7% of anti-HBc-negative children) but their third dose vaccination coverage did not differ from anti-HBc-negative children (data not shown). Most of these children are likely to be immune to HBV but may have had HBV infection, demonstrated by the presence of HBV DNA in blood or liver and being HBsAg negative [32].

In our study, 93% of children had documented evidence of hepatitis B vaccination birth dose and among those with plausible dates of administration, 92% have received the vaccine within 1 day of birth. This is only slightly below the reported administrative and official coverage of 95% for 2013 and confirms the birth dose vaccination coverage achievement of this regional hepatitis B control target [17]. Birth dose coverage and the proportion of children receiving the vaccine on the day of birth were lower in regions, where HBsAg-positive cases were found (Appendix, Table A4 and A5). Vaccination against HBV offers a very high level of protection but a minimal residual risk for infection and MTCT remains [33]. To achieve better PMTCT outcomes, the Republic of Moldova should sustain high hepatitis B birth dose coverage and introduce additional PMTCT measures: universal screening of pregnant women for HBV, timely and adequate treatment of those who have high viral loads, and administration of birth dose and hepatitis B immunoglobulin to children born to infected mothers within the first hours after birth [4,^33^,34].

The other vaccination target of the action plan is 95% coverage with three doses of hepatitis B vaccine [9]. In 2011, the Republic of Moldova switched from a 3-dose to a 4-dose vaccination schedule with three doses of pentavalent vaccine at 2, 4, and 6 months [35,36]. This change may partially explain the decline in vaccine coverage from dose two (92%) to three (86%) and some children therefore still fulfilling the definition for three doses of hepatitis B vaccine. This is supported by official data reporting coverage of 91% for 2013 [17].

In 2022, the European Technical Advisory Group of Experts on Immunization (ETAGE) considered the serosurvey results and hepatitis B vaccination coverage data of the three previous years and validated the achievement of regional hepatitis B control targets in the Republic of Moldova. The Ministry of Health should continue efforts to sustain high hepatitis B coverage and ensure equal access to hepatitis B and other routine vaccines to all children. Especially important will be to conduct catch-up vaccination through tailored campaigns to close any vaccination gaps in the population as a result of the disruption of routine immunization service delivery during the COVID-19 pandemic.

Our study has several limitations: We do not have detailed vaccination and other data from children who refused to participate in the study. However, with a high overall response rate of over 90% covering participants from almost all districts in the country, we consider our study to be adequately representative. This study did not include the autonomous region of Transnistria in the east of the Republic of Moldova, thus, the results are only applicable to the districts and municipalities of the right part of the Dniester River in the Republic of Moldova. All tests conducted in this study were done using ELISA and tests for HBV DNA were not part of the framework of this study, leaving status of anti-HBc-positive but HBsAg-negative children unclear.

## Conclusion

The successful implementation of the hepatitis B vaccination programme in the Republic of Moldova has largely reduced MTCT of HBV and infections in early childhood. The serosurvey results showed that the Republic of Moldova has achieved the 2020 outcome target for hepatitis B control in the WHO European Region. This will lead to reduced prevalence of chronic hepatitis B and subsequent sequelae and mortality in the future. After successfully reaching hepatitis B control targets, the Republic of Moldova should set a new, more ambitious goal to end hepatitis B as a public health threat in line with global and regional strategies.

Given the potential to demonstrate the impact of vaccination on hepatitis B disease burden and to validate achieving the set control target, hepatitis B serosurveys could be used as an instrument in other countries with high hepatitis B vaccination coverage to document the value of vaccination. Further studies assessing the burden of hepatitis B which has been prevented by vaccination are needed in order to reiterate the importance of vaccination programmes and the maintenance of high vaccination coverage. Sustained vaccination efforts by addressing any immunization inequity in the Republic of Moldova and high coverage with additional PMTCT measures will be crucial on the path to hepatitis B elimination.

## Declarations of competing interests

The authors alone are responsible for the views expressed in this article and they do not necessarily represent the views, decisions, or policies of the institutions with which they are affiliated.

## Appendix Supplementary materials

Image, application 1
