# Supplementary material for: Evaluating the hepatitis B vaccination impact in the Republic of Moldova: A nationwide representative serosurvey of children born in 2013
Source: IJID Reg. 2023 Nov 8;10:60–6. doi: 10.1016/j.ijregi.2023.11.003 (PMC10881275; doi:10.1016/j.ijregi.2023.11.003)
Supplement: Supplementary file 1 [file mmc1.docx]

***Appendix A. Supplementary data***

Table A1: Sample size calculation and sample distribution across study areas in the Republic of Moldova, 2020/2021

|  | **Expected prevalence** | **Upper precision bound** | **Sample size** |
| --- | --- | --- | --- |
| **Total (2 areas)** | 0.30% | 0.69% | 3,352* |
| Chisinau municipality | 0.30% | 0.99% | 1,246 |
| Remaining districts in the right part of Dniester River of the Republic of Moldova | 0.30% | 0.81% | 2,106 |
| *values used for the sample size calculations: α = 0.05; Power (1-β) = 80%; Design effect = 2 | | | |


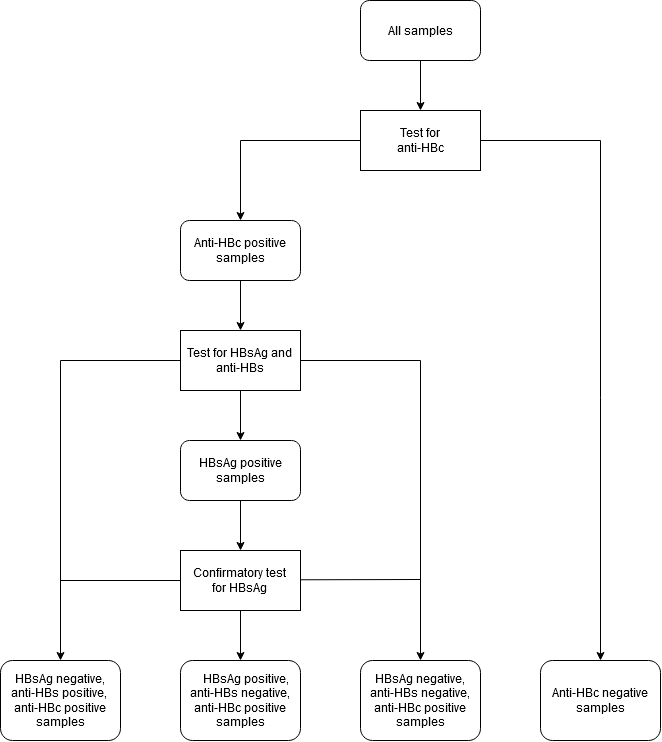


Figure A1: Laboratory testing algorithm for hepatitis B seromarkers, Republic of Moldova, 2020/2021

Table A2: Interpretation of serological test results, reproduced from WHO - Documenting the impact of Hepatitis B immunization: best practices for conducting a serosurvey (<https://apps.who.int/iris/handle/10665/70808>) [11]

| Serological test results | | | | Interpretation |
| --- | --- | --- | --- | --- |
| HBsAg | **anti-HBc (total)** | **anti-HBc (IgM)** | **anti-HBs (total)** |  |
| - | - | - | - | Susceptible; never infected |
| + | - | - | - | Early acute infection or transient after vaccination (21 days) |
| + | + | + | - | Acute infection |
| - | + | + | +/- | Acute resolving infection |
| + | + | - | +/- | Chronic infection |
| + | - | - | + | Chronic infection │ *(undetectable or false negative anti-HBc)* |
| - | + | - | + | Past infection, recovered and immune** |
| - | - | - | + | Immune due to hepatitis B vaccination † |
| - | + | - | - | Previously infected:  *Recovering from acute HBV infection*  *Previously exposed, undetectable anti-HBs*  *Current infection with undetectable HBsAg*  or Susceptible:  *Susceptible with false positive anti-HBc*  *Passive transfer to an infant from HBsAg(+) mother* |
| (-) seronegative; (+) seropositive.  ** this pattern may also be seen in successfully immunized individuals who subsequently are asymptomatically infected  † if titer >10 mIU/ml, induced trough hepatitis B vaccination or immune globulin.  Table adapted from Center for Disease Control and Prevention. National Center for HIV/AIDS, Viral Hepatitis, STD and TB Prevention. Available from Shepard et al. Epidemiol Rev 2006;28:112-25. | | | | |

Table A3: Sensitivity analysis of laboratory test results in crude analysis and stratified analysis incorporating cluster effects with residential location classification as recorded in the dataset, Republic of Moldova, 2020/2021 (n = 3,064)

| Laboratory test results | n | Crude proportion | 95% Confidence Interval | Weighted proportion | 95% Confidence Interval |
| --- | --- | --- | --- | --- | --- |
| Anti-HBc (+), anti-HBs (-), HBsAg (+) | 7 | 0.23% | 0.11–0.48% | 0.22% | 0.10–0.53% |
| Anti-HBc (+), anti-HBs (+), HBsAg (-) | 52 | 1.7% | 1.3–2.2% | 1.6% | 0.96–2.6% |
| Anti-HBc (+), anti-HBs (-), HBsAg (-) | 40 | 1.3% | 0.96–1.8% | 1.2% | 0.85–1.7% |
| Anti-HBc (-) | 2,965 | 96.8% | 96.1–97.3% | 97.0% | 95.6–97.9% |

anti-HBc: hepatitis B core antibody, anti-HBs: hepatitis B surface antibody, HBsAg: hepatitis B surface antigen.

Table A4: Analysis of weighted hepatitis B birth dose and third dose vaccination coverage, Republic of Moldova, 2020/2021

| Variable | Value | Weighted hepatitis B birth dose coverage | 95% Confidence Interval | Weighted hepatitis B third dose coverage | 95% Confidence Interval |
| --- | --- | --- | --- | --- | --- |
| Sex | Female | 93% | 89–96% | 86% | 81–90% |
|  | Male | 93% | 89–96% | 87% | 82–91% |
| Region | Chisinau | 91% | 83–96% | 83% | 73–90% |
|  | Central | 94% | 85–98% | 89% | 77–95% |
|  | North | 91% | 76–97% | 85% | 73–92% |
|  | South | 97% | 89–99% | 89% | 81–94% |
| Residential location | Rural | 96% | 92–98% | 91% | 85–95% |
|  | Urban | 92% | 85–96% | 84% | 77–89% |
| Study area | Chisinau municipality | 91% | 83–96% | 83% | 73–90% |
|  | Other areas | 94% | 89–97% | 88% | 82–92% |
| Total |  | **93%** | **89-96%** | **86%** | **82-90%** |

Table A5: Time of administration of birth dose by region, Republic of Moldova, 2020/2021

| Region | Time of administration of birth dose | | |
| --- | --- | --- | --- |
|  | On the day of birth | One day after birth | More than one day after birth |
|  | n (%) | n (%) | n (%) |
| Chisinau | 700 (76%) | 129 (14%) | 90 (9.8%) |
| Central | 542 (74%) | 133 (18%) | 59 (8.0%) |
| North | 331 (60%) | 171 (31%) | 46 (8.4%) |
| South | 393 (80%) | 66 (13%) | 32 (6.5%) |
| Total | **1,966 (73%)** | **499 (19%)** | **227 (8.4%)** |
